# Editorial for Special Issue “Bacterial Toxin-Antitoxin Systems”

**DOI:** 10.3390/microorganisms12010128

**Published:** 2024-01-08

**Authors:** Muhammad Kamruzzaman

**Affiliations:** Centre for Infectious Diseases and Microbiology, Westmead Institute for Medical Research, University of Sydney, Sydney, NSW 2145, Australia; muhammad.kamruzzaman@sydney.edu.au

Toxin antitoxin systems (TAS) are widely distributed in bacterial chromosomes as well as on mobile genetic elements. Without controversy, TAS play a key role in maintaining genetic materials [1,2]. They are also involved in various physiological activities in bacteria, including stress response, virulence, biofilm formation, antibiotic tolerance, and bacteriophage propagation [3,4,5,6]. Some of the functions of TAS have some degree of controversy; however, growing research in this area has shown the importance of TAS in bacterial lifestyle [7,8,9,10].

In this special issue, we tried to compile findings on the identification and characterisation of novel TAS, the biological functions of TAS, and the applications of TAS in biotechnology and medicine. A manuscript by Choi et al. (contribution 2 in the special issue) describes the identification, genetic and functional characterisation of the *mazEF*-type TA in the pathogenic bacterium *Agrobacterium tumefaciens*.

Manuscript by Schirmer et al. [11] (contribution 1) demonstrated the evolutionary diversification of *Bartonella* effector proteins (Beps) from a single ancestral FicTA toxin-antitoxin module. It showed how FicA-like BiaA antitoxin can interfere with the effector functions of Beps in *Bartonella* by forming a tight complex with Beps. Extensive analysis using multiple tools demonstrated the remarkable functional and regulatory plasticity of Beps that occur due to minor structural changes of the FIC fold.

It has been previously demonstrated that the expression of toxin *tisB* of *tisB*/*istR*-*1* TA system is induced in response to SOS signal and the role of *tisB*/*istR*-*1* TA system in forming persister cells [12]. A manuscript by Edelmann et al. (contribution 4) demonstrated that this SOS-dependent tisB toxin activation and persister cell formation is conditional and depends on the DNA-damaging agents.

TAS have been used previously in several biotechnological approaches, including constructing cloning vectors, stable expression vectors, and producing proteins [13,14]. In this issue, the authors in contribution 3, developed a novel method for the scarless deletion of a gene of interest in bacteria by using *Vibrio parahaemolyticus* YoeB toxin as a counter-selectable marker.

A very comprehensive review on type II TA systems [15] (contribution 5) compiled experimentally demonstrated bonafide biological functions of type II TAS in bacteria, including the role of TAS in the maintenance of genetic materials, bacterial virulence and pathogenesis, biofilm formation, contribution to bacterial resistance to bacteriophages, bacteriophage propagation in host bacteria, different stress responses and discussed the application of TAS in biotechnology and medicine. Several limitations in the TA research area have also been discussed in this review.

The increasing interest in TA research is now elucidated by continuously discovering new TA systems with novel functions and their structure-function relationships. Still, more research needs to understand the role of so many uncharacterised TAS. Many TA systems or their close relatives have been identified in bacterial chromosomes and on different mobile genetic elements, such as on plasmids. More research is needed to understand the crosstalk between those TAS. TAS present on MGE can move to other bacterial species, but it is not very well-known whether these TAS play the same functions in diverse bacterial species or are conserved to specific bacteria [16]. Several studies identified orphan toxin or antitoxin genes in bacterial chromosomes and on different mobile genetic elements [17,18]. A question arises: what is the function of these lone toxins or antitoxins? It was demonstrated that orphan antitoxin or antitoxin-like genes present in bacteriophage genomes provide an advantage to bacteriophage infection by protecting phage DNA from bacterial endonuclease-type toxins [19,20]. More research is needed to understand the function of lone toxin or antitoxin, specifically when they are present on plasmids or phage genomes. Orphan antitoxins may provide advantages to the mobile elements for their dissemination by protecting them from the bacterial defence mechanism immediately after transfer to a new host.

## References

[1] Hayes F. (2003). Toxins-antitoxins: Plasmid maintenance, programmed cell death, and cell cycle arrest. Science.

[2] Huguet K.T., Gonnet M., Doublet B., Cloeckaert A. (2016). A toxin antitoxin system promotes the maintenance of the IncA/C-mobilizable Salmonella Genomic Island 1. Sci. Rep..

[3] Unterholzner S.J., Poppenberger B., Rozhon W. (2013). Toxin-antitoxin systems: Biology, identification, and application. Mob. Genet. Elem..

[4] Van Melderen L. (2010). Toxin-antitoxin systems: Why so many, what for?. Curr. Opin. Microbiol..

[5] Wen Y., Behiels E., Devreese B. (2014). Toxin-Antitoxin systems: Their role in persistence, biofilm formation, and pathogenicity. Pathog. Dis..

[6] Yamaguchi Y., Park J.H., Inouye M. (2011). Toxin-antitoxin systems in bacteria and archaea. Annu. Rev. Genet..

[7] Jurenas D., Fraikin N., Goormaghtigh F., Van Melderen L. (2022). Biology and evolution of bacterial toxin-antitoxin systems. Nat. Rev. Microbiol..

[8] LeRoux M., Laub M.T. (2022). Toxin-Antitoxin Systems as Phage Defense Elements. Annu. Rev. Microbiol..

[9] Pizzolato-Cezar L.R., Spira B., Machini M.T. (2023). Bacterial toxin-antitoxin systems: Novel insights on toxin activation across populations and experimental shortcomings. Curr. Res. Microb. Sci..

[10] Qiu J., Zhai Y., Wei M., Zheng C., Jiao X. (2022). Toxin-antitoxin systems: Classification, biological roles, and applications. Microbiol. Res..

[11] Schirmer T., de Beer T.A.P., Tamegger S., Harms A., Dietz N., Dranow D.M., Edwards T.E., Myler P.J., Phan I., Dehio C. (2021). Evolutionary Diversification of Host-Targeted Bartonella Effectors Proteins Derived from a Conserved FicTA Toxin-Antitoxin Module. Microorganisms.

[12] Dorr T., Vulic M., Lewis K. (2010). Ciprofloxacin causes persister formation by inducing the TisB toxin in *Escherichia coli*. PLoS Biol..

[13] Stieber D., Gabant P., Szpirer C. (2008). The art of selective killing: Plasmid toxin/antitoxin systems and their technological applications. Biotechniques.

[14] Suzuki M., Zhang J., Liu M., Woychik N.A., Inouye M. (2005). Single protein production in living cells facilitated by an mRNA interferase. Mol. Cell.

[15] Kamruzzaman M., Wu A.Y., Iredell J.R. (2021). Biological Functions of Type II Toxin-Antitoxin Systems in Bacteria. Microorganisms.

[16] Wu A.Y., Kamruzzaman M., Iredell J.R. (2020). Specialised functions of two common plasmid mediated toxin-antitoxin systems, *ccdAB* and *pemIK*, in *Enterobacteriaceae*. PLoS ONE.

[17] Akarsu H., Bordes P., Mansour M., Bigot D.J., Genevaux P., Falquet L. (2019). TASmania: A bacterial Toxin-Antitoxin Systems database. PLoS Comput. Biol..

[18] Leplae R., Geeraerts D., Hallez R., Guglielmini J., Dreze P., Van Melderen L. (2011). Diversity of bacterial type II toxin-antitoxin systems: A comprehensive search and functional analysis of novel families. Nucleic Acids Res..

[19] Alawneh A.M., Qi D., Yonesaki T., Otsuka Y. (2016). An ADP-ribosyltransferase Alt of bacteriophage T4 negatively regulates the *Escherichia coli* MazF toxin of a toxin-antitoxin module. Mol. Microbiol..

[20] Otsuka Y., Yonesaki T. (2012). Dmd of bacteriophage T4 functions as an antitoxin against *Escherichia coli* LsoA and RnlA toxins. Mol. Microbiol..

